# Molecular developmental evidence for a subcoxal origin of pleurites in insects and identity of the subcoxa in the gnathal appendages

**DOI:** 10.1038/srep15757

**Published:** 2015-10-28

**Authors:** Joshua F. Coulcher, Gregory D. Edgecombe, Maximilian J. Telford

**Affiliations:** 1 Laboratoire de Biologie Intégrative des Organismes Marins (BIOM), Observatoire Océanologique de Banyuls, Avenue du Fontaulé, 66650 Banyuls-sur-mer, France; 2 Department of Earth Sciences, The Natural History Museum, London SW7 5BD, United Kingdom; 3 Department of Genetics, Evolution and Environment, Darwin Building, Gower St, London WC1E 6BT, United Kingdom

## Abstract

Pleurites are chitinous plates in the body wall of insects and myriapods. They are believed to be an adaptation to locomotion on land but their developmental and evolutionary origins are unclear. A widely endorsed explanation for their origin is through toughening pre-existing parts of the body wall; in contrast, the subcoxal theory suggests pleurites derive from a redeployment of the proximal-most section of the leg, the subcoxa. Here, by studying expression of appendage patterning genes in embryos and larvae of the beetle *Tribolium castaneum*, we provide the first molecular evidence for the existence of a cryptic subcoxal segment in developing legs. We follow this structure during development and show that the embryonic subcoxa later forms the pleurites of the larva as predicted by the subcoxal theory. Our data also demonstrate that subcoxal segments are present in all post-antennal appendages, including the first molecular evidence of a two-segmented mandible with a subcoxal segment in insects.

Members of only three animal phyla—chordates, molluscs and arthropods - are fully adapted to living in terrestrial environments. This move to land has necessitated the evolution of a series of traits to cope with the novel environment such as air breathing, prevention of desiccation and new ways of mating. Here we consider the evolutionary history of insect pleurites - an adaptation that allows locomotion on land despite the drastic reduction in buoyancy provided by air compared to water. The evolution of this essential terrestrial adaptation has a direct parallel in the evolution of the pelvic girdle in land vertebrates.

Pleurites are chitinous plates found as part of the lateral body wall (the pleuron) of arthropods. They vary in form from small and sometimes numerous sclerotized plates and arches surrounding the leg base as found in the apterygote (wingless) insects (see Fig. 1a), to a single fused lateral plate typical of the pterygote (winged) insects (see Fig. 1b). Pleurites provide stability and structural rigidity to the base of the leg and the lateral body wall and are important for activities like walking without the support of water and for burrowing. In flying insects (Pterygota), pleurites strengthen the wing-bearing thoracic segments further by extending to the base of the wing. Pleurites are ubiquitous in two terrestrial arthropod groups, the hexapods and myriapods, but are lacking in most crustaceans and from the other lineage of predominantly terrestrial arthropods, the chelicerates^1^,^2^,^3^.

One view for the evolutionary and ontogenetic origin of insect pleurites, championed by Sidnie Manton, argues that they derive from within the pleuron itself. In Manton’s theory, the pleural sclerites are regions of the pleuron that evolved by hardening in response to the particular physical stresses caused by different forms of terrestrial locomotion, including flight, and by activities such as burrowing through soil, enrolment and crawling through rock crevices^2^,^4^.

The subcoxal theory, in contrast, originates in the work of nineteenth century biologists^5^. Heymons was the first to hypothesize the existence, in a hemipteran, of a proximal subdivision of the embryonic leg which he labelled the subcoxa. Heymons proposed that pleurites develop from this subcoxal leg segment (see Fig. 1d)^6^. Snodgrass extended Heymons’ ideas of subcoxal development and argued that the proposed ontogenetic origin of pleurites recapitulated evolutionary history and that pleurites evolved from an ancestral subcoxal segment (see Fig. 1c,d)^1^,^7^. Recently there has been revived interested in the subcoxal theory, with supporting morphological evidence from embryology and from muscle insertion patterns^3^,^8^,^9^,^10^,^11^,^12^.

In the modern form of the subcoxal theory, pleurite rings surrounding the leg base (which may be fragmented or incomplete in different hexapod and myriapod lineages) are identified as a trochantinopleurite and eupleurite^3^. They are either considered both to be derivates of the subcoxa or to represent a subcoxa and precoxa, respectively^13^.

To investigate the evidence for the existence of a subcoxal leg segment in hexapods and its potential involvement in forming pleurites, we studied the expression of molecular markers of appendage development in developing embryos of the red flour beetle *Tribolium castaneum*. *Tribolium* has pleurites typical of holometabolous insects and is a good animal in which to study their development as the pleurites are unfragmented, relatively large and more segment-like in the larval stages than in some other hexapods^3^.

To identify the subcoxa, we required markers that could define subdivisions within the developing legs and markers that could then identify the subdivisions identified in this way. Joint formation in both *Drosophila* and *Tribolium* appendages is controlled by Notch signalling and involves multiple genes including *serrate* (*ser*) and *Delta*^14^,^15^,^16^; the process appears to be conserved across Arthropoda^17^. We chose to study the expression of the *Tribolium* homologue of the gene *serrate (Tc-ser)* because *Tc-ser* expression proved significantly easier to detect by *in situ* hybridization than *Notch* (data not shown). *ser* is a transmembrane ligand of the Notch receptor and regulates Notch activation in adjacent cells. It is expressed in a ring of cells on the distal part of each appendage segment and is therefore expressed slightly proximal to where the segment boundary will form^15^. Notch signalling is regulated by ‘leg gap genes’ in *Drosophila* that pattern the proximo-distal (PD) axis, such as *homothorax (hth), dachshund (dac) and Distal-less (Dll)*. The expression and function of leg gap genes are conserved, with slight differences, across arthropod leg development^15^,^16^,^18^,^19^.

## Results and Discussion

**Figure 2:**
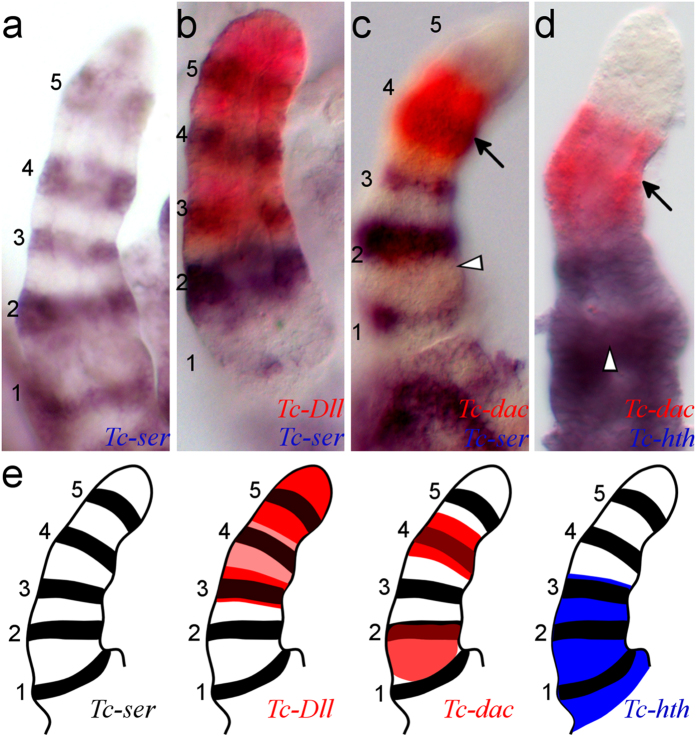
Defining *Tc-ser* expression domains in the embryonic leg relative to limb gap gene expression. Gene expression was detected by *in situ* hybridisation of dissected legs from germ band-retracted stage embryos. All views are distal to the top and lateral to the left. Domains of *Tc-ser* are numbered 1 to 5 from proximal to distal. The distal domain of *Tc-dac* is indicated with an arrow, proximal domain of *Tc-dac* is indicated with a white arrowhead (in (**c**,**d**)). (**a**) Expression of *Tc-ser* (blue). There are five domains of *Tc-ser* expression in the embryonic leg. (**b**) Expression of *Tc-ser* (blue) and *Tc-Dll* (red). *Tc-Dll* is co-expressed with the third, fourth and fifth domains of *Tc-ser*, but is most strongly expressed in two domains, a ‘ring’ and a ‘sock’ domain. The ring domain is co-expressed with the third *Tc-ser* domain, the sock domain is co-expressed with the fifth domain. (**c**) Expression of *Tc-ser* (blue) and *Tc-dac* (red). *Tc-dac* expression shows proximal and distal expression domains. The distal domain of *Tc-dac* is co-expressed with the fourth domain of *Tc-ser* whilst the proximal domain of *Tc-dac* expression is expressed slightly overlapping and proximal to the second *Tc-ser* domain (see **b,d**). (**d**) Expression of *Tc-hth* (blue) and *Tc-dac* (red). (**e**) Diagrams showing the gene expression of *Tc-Dll, Tc-dac and Tc-hth* relative to *Tc-ser* shown in (**a**,**d**). The distal limit of *Tc-hth* expression can be related to *Tc-ser* expression by comparison with *Tc-dac* expression. *Tc-hth* is expressed adjacent to the distal domain of *Tc-dac* expression. *Tc-hth* is therefore co-expressed with the first, second and third domains of *Tc-ser.*

We found five domains of *Tc-ser* expression in the embryonic leg rudiment (see Fig. 2a), corresponding to five joints separating six segments. As the *Tribolium* larval leg has five ‘true’ leg segments (the coxa, trochanter, femur, tibiotarsus and pretarsus^16^) the presence of an extra embryonic segment led us to infer that the proximal-most segment might be the subcoxa. To prove this, we aimed to determine which segments these domains of *Tc-ser* expression correspond to in the larval leg.

The proximal-most *Tc-ser* domain is co-expressed with *Tc-hth*, the second with both *Tc-hth* and the proximal domain of *Tc-dac*^20^, whereas the third domain has both *Tc-hth* and *Tc-Dll* co-expression. The fourth *Tc-ser* domain is co-expressed with the distal domain of *Tc-dac* and *Tc-Dll* and finally, the fifth is co-expressed only with *Tc-Dll* (see Fig. 2b–e). The proximal domain of *Tc-dac* is faintly expressed at this stage, and may also be expressed in the proximal-most *Tc-ser* domain (see below).

**Figure 3:**
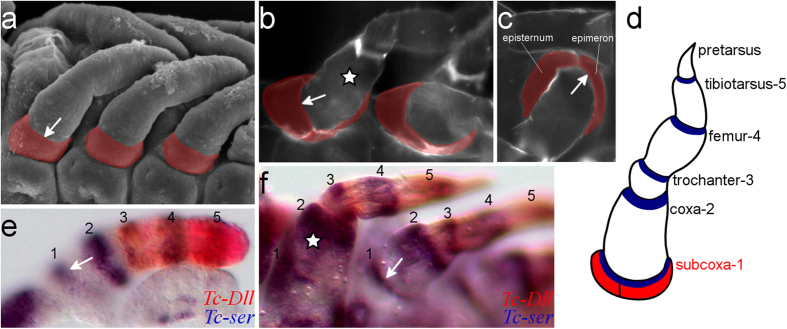
The subcoxal segment of the embryonic leg becomes pleurites in the larva. The white arrow indicates the boundary between the subcoxal and the coxal segments in (**a**–**c**) and (**e**,**f**). A white star indicates the coxa in b and f. (**a**) Scanning electron micrograph (SEM) of developing leg appendages of *Tribolium* embryos showing the presence of a subcoxa (highlighted red) on the developing leg. Embryo at germ band retracting stage, ventral-lateral view. (**b**) Cuticle preparation of a first instar larva, ventral view. The pleurites are highlighted in red. (**c**) Cuticle preparation of a first instar larva, lateral view. The two pleural sclerites typical of ectognathous insects, the epimeron and episternum, are indicated (highlighted in red). This condition is typical of pterygote insects. (**d**) Schematic representation of a *Tribolium* larval leg with the name of the limb segments and their corresponding domains of *Tc-ser* expression (in blue) in the distal part of each segment. The pleurites are highlighted in red. (**e**) *in situ* hybridisation with expression of *Tc-ser* (blue) and *Tc-Dll* (red) in a dissected leg of a germ band retracted stage embryo. (**f**) *in situ* hybridisation with expression of *Tc-ser* (blue) and *Tc-Dll* (red) in a late stage embryo undergoing dorsal closure.

As the coxa is both the first typical leg segment and is easy to identify by virtue of its large size and characteristic conical shape, we first tried to identify the domain of *Tc-ser* that corresponds to this segment. To do this, we followed *Tc-ser* expression and *Tc-Dll* expression through to later stage embryos in which leg morphology is more similar to larval leg morphology (see Fig. 3).

Comparison of the morphology of the larval leg to that of the morphology of late stage embryos shows that the coxa (indicated with a star in Fig. 3b,f) corresponds to the second segment as identified by *Tc-ser* expression in the embryonic leg: the coxa lacks *Tc-Dll* expression and has the second domain of *Tc-ser* expressed at its distal border whereas *Tc-Dll* is co-expressed with the third *Tc-ser* domain, which thus identifies the latter as the third leg segment, the trochanter. Having established the identity of the coxa and trochanter, the five typical leg segments (coxa, trochanter, femur, tibiotarsus and pretarsus) are seen to correspond to the second, third, fourth, fifth and sixth segments of the embryonic leg (marked by *Tc-ser* expression).

Significantly, a *Tc-ser* domain is identified proximal to the coxal domain (arrow in Fig. 3e,f). Therefore we define the *Tc-ser* domains in proximo-distal order as the subcoxa-1 domain, the coxa-2 domain, the trochanter-3 domain, the femur-4 domain, and the tibiotarsus-5 domain, respectively (see Fig. 3d). The pretarsal segment is distal to the tibia *Tc-ser* domain-5. In *Tribolium* first instar larvae, the pleurites are flattened radially on the body wall and support the base of the coxa (the largest segment of the leg) (see star in Fig. 3b,f). These sclerites are partly fused to the body wall and form two plates, the episternum and the epimeron, separated by the pleural suture, which is typical of holometabolous insects (see Fig. 3c). This structure develops proximally, adjacent to the coxa, and in the same position as the first domain of *Tc-ser* expression in the embryonic leg (see Fig. 3f). This demonstrates that the subcoxal segment becomes the pleurites in the larva.

**Figure 4:**
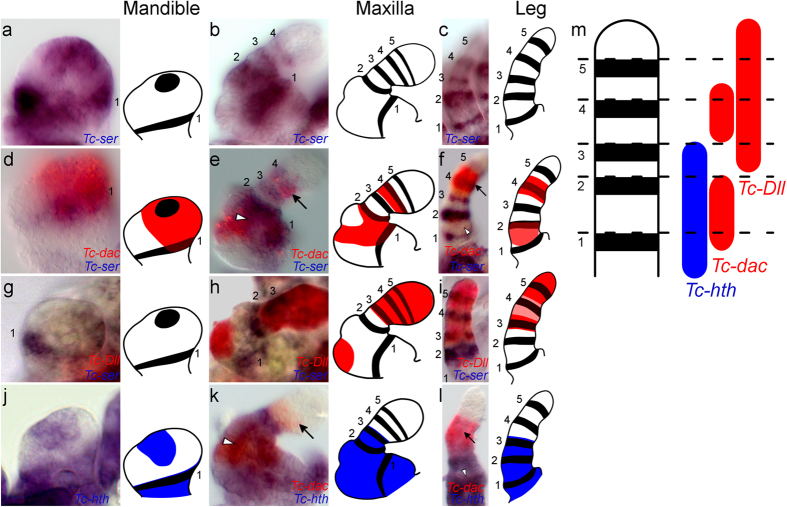
Serial homology of the subcoxa and coxal segment shown by leg gap gene and *Tc-ser* expression in the embryonic mandible, maxilla and leg. All views are distal to the top and lateral to the right except when otherwise indicated. Domains of *Tc-ser* are numbered 1 to 5 from proximal to distal. The black arrow shows the distal domain of *Tc-dac* expression, and the white arrowhead shows the expression of the proximal domain of *Tc-dac* expression. Gene expression is shown schematically for each panel. Gene expression is shown for the mandible (in a,d,g and j), the maxilla (in b,e,h and k) and leg (in c,f,i and l). (**a**) Expression of *Tc-ser* (blue) in the mandible. Lateral view. (**b**) Expression of *Tc-ser* (blue) in the maxilla. (**c**) Expression of *Tc-ser* (blue) in the leg. Lateral is to the left. (**d**) Expression of *Tc-ser* (blue) and *Tc-dac* (red) in a dissected mandible. (**e**) Expression of *Tc-ser* (blue) and *Tc-dac* (red) maxilla. (**f**) Expression of *Tc-ser* (blue) and *Tc-dac* (red) in a leg. Lateral is to the left. (**g**) Expression of *Tc-ser* (blue) and *Tc-Dll* (red) in a mandible. (**h**) Expression of *Tc-ser* (blue) and *Tc-Dll* (red) in a maxilla. There is an endite expression domain of *Tc-Dll* in the second segment, which is not present in the legs. (**i**) Expression of *Tc-ser* (blue) and *Tc-Dll* (red) in a leg. Lateral is to the left. (**j**) Expression of *Tc-hth* (blue) and *Tc-dac* (red) in a dissected mandible. (**k**) Expression of *Tc-hth* (blue) and *Tc-dac* (red) in a maxilla. The distal limit of *Tc-hth* expression in the maxilla can be related to *Tc-ser* expression by comparison with *Tc-dac* expression. *Tc-hth* is expressed up to the trochanteral-3 domain of *Tc-ser* expression and therefore co-expressed with the third *Tc-ser* domain in the maxilla and legs. (**l**) Expression of *Tc-hth* (blue) and *Tc-dac* (red) in a leg. Lateral is to the left. (**m**) Diagram showing leg gap gene expression corresponding to domains of *Tc-ser* expression.

Having shown the existence of a subcoxa in the walking appendages of *Tribolium*, we looked for homologs of this segment in other limbs by comparing expression of *Tc-ser* and the leg gap genes between the proximal segments of the legs and the gnathal appendages of *Tribolium* (see Fig. 4, Supplementary Figs S1–S5). This revealed that *Tc-ser* is expressed in ring domains along the proximal-distal axis of all appendage types (see Fig. 4a–c and Supplementary Figs S1 and S4).

The hexapod mandible is an unsegmented appendage in both the larva and the adult, however, recent morphological studies have suggested the presence of both a subcoxa and coxa in the developing mandible, and that this may be a general character of Hexapoda^9^,^11^,^21^,^22^,^23^ as well as in Myriapoda^24^. The *Tribolium* mandible limb bud shows two domains of *Tc-ser* expression - a ring and spot domain (see Fig. 4a and Supplementary Fig. S1). The spot domain is present on the distal tip of the mandibular limb bud, more specifically on the outer lobe of the developing endite that relates to the developing incisor process^25^. However, the expression of the subcoxa-1 domain of *Tc-ser* in the *Tribolium* mandible (see Fig. 4a, Supplementary Fig. S5) provides the first molecular evidence for the existence of a mandibular subcoxa.

Comparison of leg gap gene expression between the mandible, maxilla and legs shows similar expression patterns relative to *Tc-ser* expression domains (see Fig. 4m for a summary figure). For the mandible, maxilla and leg, the second segment is characterized by both *Tc-hth* and *Tc-dac* co-expression (see Fig. 4d–f,j–l). The first *Tc-ser* domain is co-expressed with *Tc-hth* (see Fig. 4j–l) and *Tc-dac* in the mandible and maxilla. *Tc-dac* is co-expressed in the lateral part of the *Tc-ser* subcoxal-1 domain in the mandible and maxilla (see arrowhead in Supplementary Figs S2 and S3), and in the leg subcoxal domain during early leg development (see Supplementary Figs S4 and S5).

We found additional support for serial homology of the subcoxa and coxa in the simultaneous activation of *Tc-ser* and *Tc-dac* in all post-antennal appendages (see Supplementary Figs S4 and S5). More evidence for the serial homology of the subcoxa of the mandible, maxilla and labium is provided by the expression of *Tc-paired* (*Tc-prd*), which marks the position of the developing endites^25^. *Tc-prd* is expressed in the coxal segment and is excluded from the subcoxal segment in the mandible, maxilla and labium (see Supplementary Fig. S6).

Taking all this evidence as a whole, we conclude that the leg subcoxa is serially homologous to the proximal-most segments of the post-antennal gnathal appendages, specifically to the cardo of the maxilla, the mandibular subcoxa and the labial postmentum.

Our results show that the pleurites likely develop from a leg segment in *Tribolium* in accordance with the subcoxal theory. Regarding pleurites in myriapods, it has been postulated that they are homologous with those of hexapods because these taxa were thought to be closely related in a group called the Atelocerata^3^. It is now widely accepted that the hexapods derive instead from within a wider pancrustacean group that does not include the myriapods. Most crustaceans lack pleurites, and the exceptions involve groups that have been proposed as likely hexapod relatives, remipedes^26^ and malacostracans. Remipedes have a detached fragment of the coxa that forms an anterior pleurite^3^ and the pleopods of malacostracans have a pleurite-like coxal sclerotization^27^. Irrespective of whether or not these are homologous with pleurites in hexapods, the absence of pleurites in other crustaceans implies convergent evolution of pleurites of hexapods and myriapods. Whether this is an adaptation to terrestrial locomotion in both is contingent on the affinities of the aquatic Remipedia. It remains to be demonstrated whether pleurites develop from a subcoxal segment in myriapods.

Understanding their precise segmental composition is necessary to understand the evolution of insect appendages from the common ancestor of hexapods and crustaceans. Regarding the evolution of the pleurites of hexapods, their absence in most crustaceans renders it likely that the insect subcoxa is homologous to one of the proximal crustacean leg segments, which typically do not integrate into the body wall. As there is significant diversity of crustacean limbs in terms of segment number and morphology, together with the fact that crustacean phylogenetic relationships relative to the hexapods remain unresolved^28^, it is currently difficult to postulate a homologous segment to the insect subcoxa and coxa. Comparative morphology argues that the insect subcoxa may be homologous to the crustacean ‘coxa’, and that the insect coxa is therefore homologous to the crustacean ‘basis’^3^,^12^. The correspondences noted above in remipede and malacostracan coxal fragments and subcoxally-derived insect pleurites are consistent with this hypothesis.

Despite the great diversity of insect limb morphology, the evidence for serially homologous limb segments suggests a shared regulatory logic for patterning limb segments within the same species. This similarity of gene regulation probably reflects common ancestry of these segments from an ancestral limb; so that, for example, the subcoxa of the mandible, maxilla, and legs could have evolved from the same leg segment in identical ancestral serially homonomous limbs. The Cambrian fossil record reveals that many arthropod taxa, such as trilobites, had a series of morphologically scarcely undifferentiated post-antennal biramous limbs^29^,^30^,^31^. Based on these facts, a scenario of insect appendage evolution from ancestral serially homologous limbs is depicted in Supplementary Fig. S7.

Our observations of development of the subcoxal segment may also contribute to understanding of the evolution of insect wings. Two main hypotheses for the origin of wings are gill theory and paranotal theory^12^,^32^,^33^. In gill theory, the wings evolved from gills (or exites), which were present on a proximal (possibly subcoxal) segment. As we have identified the precise position of the developing subcoxa in the *Tribolium* embryo, this could help to identify cells from the subcoxal segment that might contribute to the development of the wing, and show whether genes important for patterning wings (such as *vestigial)* are expressed in cells of the subcoxal segment^34^,^35^.

## Materials and Methods

### *Tribolium castaneum* culture and experimental methods

All experiments, including details of *Tribolium castaneum* culture and methods for studying gene expression in *Tribolium* embryos (whole-mount *in situ* hybridisation), techniques for studying embryo and larval morphology (scanning electron microscopy and larval cuticle preparation) were performed as previously described^25^.

Specifically regarding *in situ* hybridization, both single stainings with nitro blue tetrazolium/5-bromo-4-chloro-3-indolyl phosphate (NBT/BCIP) and double stainings (NBT/BCIP and FastRed) were performed as previously described^36^ but with slight changes to the frequency and duration of washes introduced from alternative *in situ* hybridisation protocols^37^ to reduce background staining.

Double *in situ* hybridizations were performed by adding two probes (a digoxygenin (DIG) labelled probe and a Fluorescein (FITC) labelled probe) to the embryos simultaneously at a concentration of 0.1–5% (v/v) to hybridization solution (50% Formamide (v/v), 5X SSC (750 mM Sodium Chloride , 75 mM Sodium Citrate), 0.2% (w/v) Salmon sperm DNA, 0.1% (w/v) Heparin, 0.02% Tween-20, adjusted to pH6.5). Hybridisation was performed at 60 °C for 42 hours.

An alkaline phosphatase conjugated anti-DIG antibody was incubated with the embryos either overnight at 4 °C or for one hour at room temperature. The first staining was performed with NBT/BCIP. After the staining was completed, the alkaline phosphatase conjugated anti-DIG antibody was inactivated by denaturing the protein for 15 minutes at 65 °C in hybridization solution with 0.3% (w/v) Sodium Dodecyl Sulphate (SDS). A second staining was performed by first incubating the embryos with alkaline phosphatase conjugated anti-FITC antibody and then subsequently staining with FastRed (Sigma).

### Cloning of *Tribolium* genes

Genes were cloned in order to synthesize antisense labelled RNA probes to detect gene expression by *in situ* hybridisation. *Tc-Dll*, *Tc-hth*, *Tc-ser* and *Tc-prd* were amplified from cDNA by PCR amplification using the following primers: *Tc-ser* (fw: 5′- AAGGCAACGTTTGCCAATTCGG-3′ and rv: 5′-TCCCATGTGCAACTTCCTGGAGAT-3′, fw: 5′-TCCTTCTGCTACTCAACCTGCTAC-3′ and rv: 5′-GGGGACATTCGCACTTGAACAT-3′, fw: 5′- ATTTGGTGCGGTCTGGGAAACT -3′ and rv: 5′-TCGGGGTTTTGCGCTTTGTAGA-3′). Three sections of *Tc-ser* were amplified and cloned to provide more than 2.5 kb of gene specific sequence to increase the strength of signal of gene expression in whole-mount *in situ* hybridisation experiments. *Tc-Dll* (fw: 5′-CAGCAGGTGCTCAATGTGTT-3′ and rv: 5′-ATTAAACAGCTGGCCACACC-3′), *Tc-prd* (fw: 5′-ATGCACAGACATTGCTTTGG-3′ and rv: 5′-GGATCGTCACAGTGTTGGTG-3′), *Tc-hth* (fw: 5′-AGCCGTTTTCTCCAAACAGA-3′ and rv: 5′-GGATAGTGCGCGTACTGGTT-3′). Accession numbers are as follows: *Tc-ser* (GenBank: XM_008198074.1), *Tc-Dll* (GenBank: NM_001039439), *Tc-hth* (GenBank: NM_001039400), and *Tc-prd* (GenBank: NM_001077622).

## Additional Information

**How to cite this article**: Coulcher, J. F. *et al.* Molecular developmental evidence for a subcoxal origin of pleurites in insects and identity of the subcoxa in the gnathal appendages. *Sci. Rep.* **5**, 15757; doi: 10.1038/srep15757 (2015).

## Supplementary Material

Supplementary Information

## Figures and Tables

**Figure 1:**
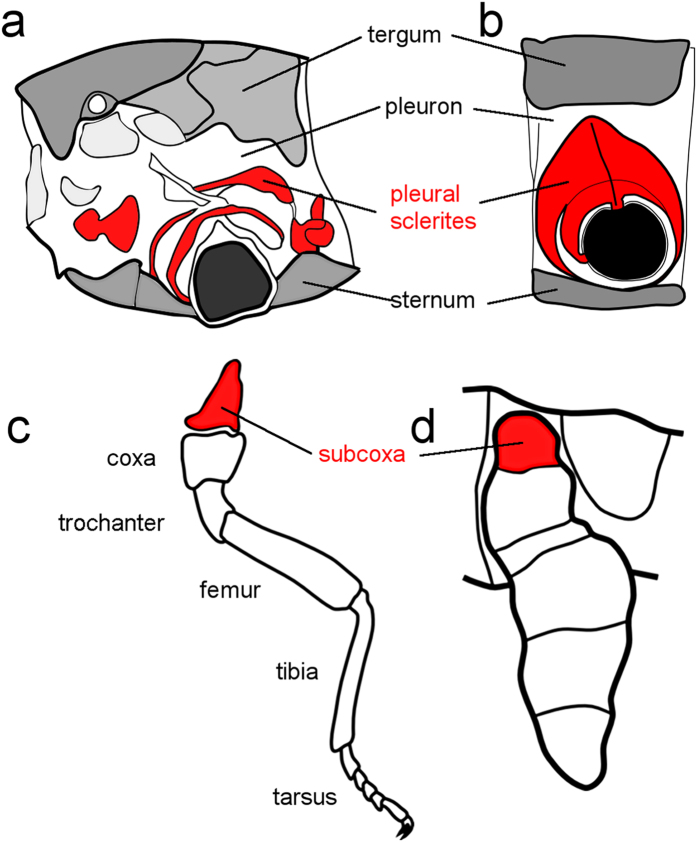
The subcoxal theory of the origin of the pleural sclerites. The classic subcoxal theory states that the pleurites on the lateral insect thorax develop from an embryonic subcoxal segment. (**a**) Schematic showing the pleurites (red) in the mesothorax of a proturan, *Eosentomon germanicum*. (**b**) Diagram of the basic structure of a pterygote insect pleuron on a wingless thoracic segment. For clarity, the limb more distal to the subcoxa is not shown in (**a**,**b**). (**c**) Schematic showing segmental identities in a generalized embryonic limb. The subcoxa (which forms the pleural sclerites) is highlighted in red. (**d**) Schematic of the subcoxa (red) in an embryonic limb of *Naucoris* sp. (Rhynchota, Hemiptera). (**a**–**c**) were adapted from^1^ (**d**) was adapted from^6^.
