# Supplementary material for: Molecular developmental evidence for a subcoxal origin of pleurites in insects and identity of the subcoxa in the gnathal appendages
Source: Sci Rep. 2015 Oct 28;5:15757. doi: 10.1038/srep15757 (PMC4623811; doi:10.1038/srep15757)
Supplement: Supplementary Information [file srep15757-s1.pdf]

Molecular developmental evidence for a subcoxal origin of pleurites in insects and identity of the subcoxa in the gnathal appendages

Supplementary Information

Joshua F. Coulcher[1], Gregory D. Edgecombe[2] and Maximilian J. Telford[3]

[1] Laboratoire de Biologie Intégrative des Organismes Marins (BIOM), Observatoire Océanologique de Banyuls, Avenue du Fontaulé, 66650 Banyuls-sur-mer, France; email: joshua.coulcher@obs-banyuls.fr

[2] Department of Earth Sciences, The Natural History Museum, London SW7 5BD, United Kingdom; email: g.edgecombe@nhm.ac.uk

[3] Department of Genetics, Evolution and Environment, Darwin Building, Gower St, London WC1E 6BT, United Kingdom; email: m.telford@ucl.ac.uk

Supplementary figure S1

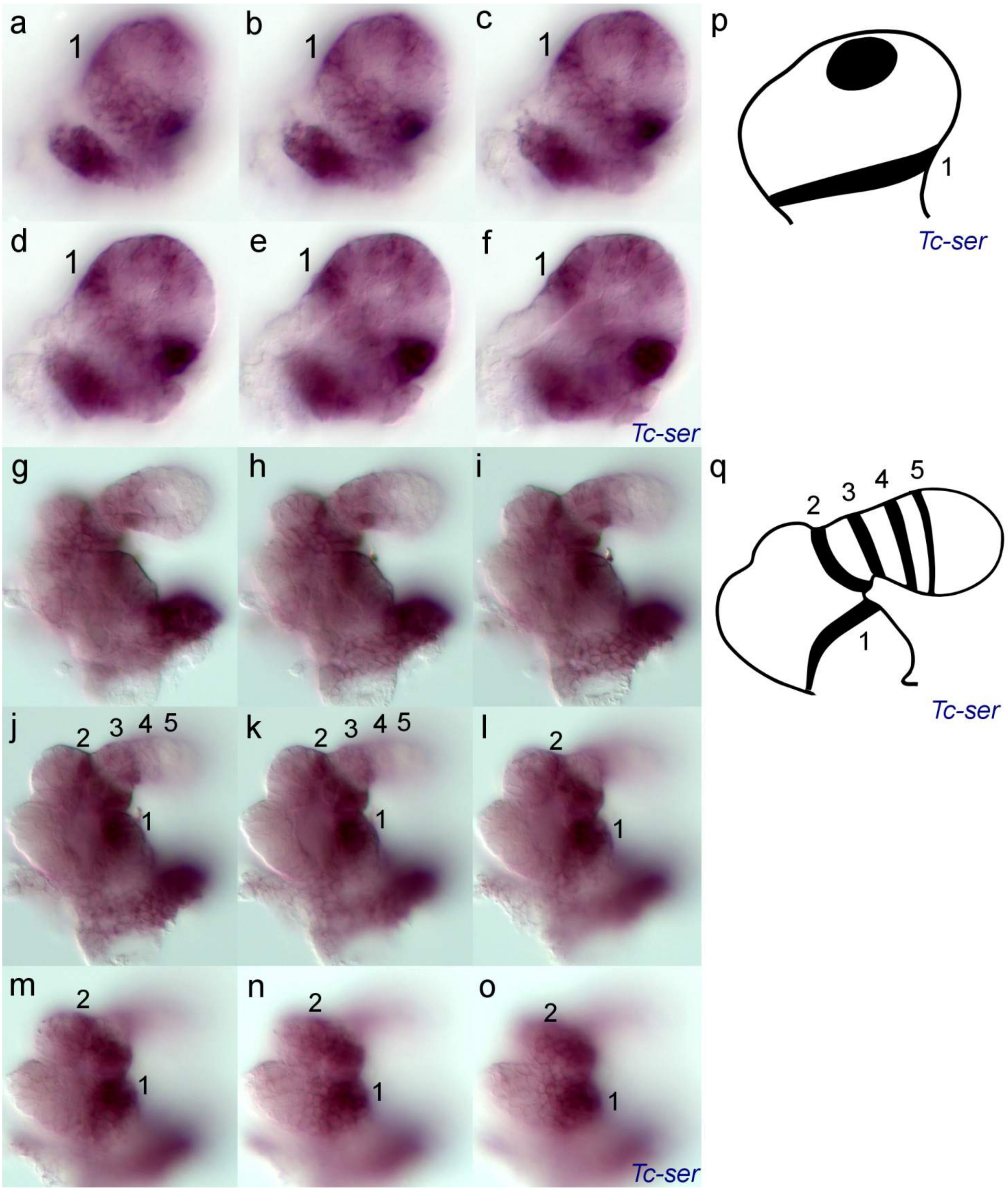

Supplementary figure S1: Montage of images of *Tc-ser* expression in a dissected mandible and maxilla with increasing depth of focus along the z axis. All views are ventral with anterior to the left. *Tc-ser* (blue) gene expression was detected by *in situ* hybridisation. All views of the mandible are lateral with distal on top. (a-f). The diagram of the mandible in (p) is lateral on the right, medial on the left and distal on top. All views of the maxilla are lateral on the right, medial on the left and distal on top. The plane of focus is highest in (a) for the mandible and (g) for the maxilla. The focal plane incrementally descends through the dissected mandible from (a) through (f) and through the maxilla from (g) through (o). Tc-ser domains that mark segmental development are indicated with numbers where they are clear and that region of the ectoderm is in focus. *Tc-ser* gene expression is summarized in the schematic of a mandible in (p) and a maxilla (q).

Supplementary figure S2

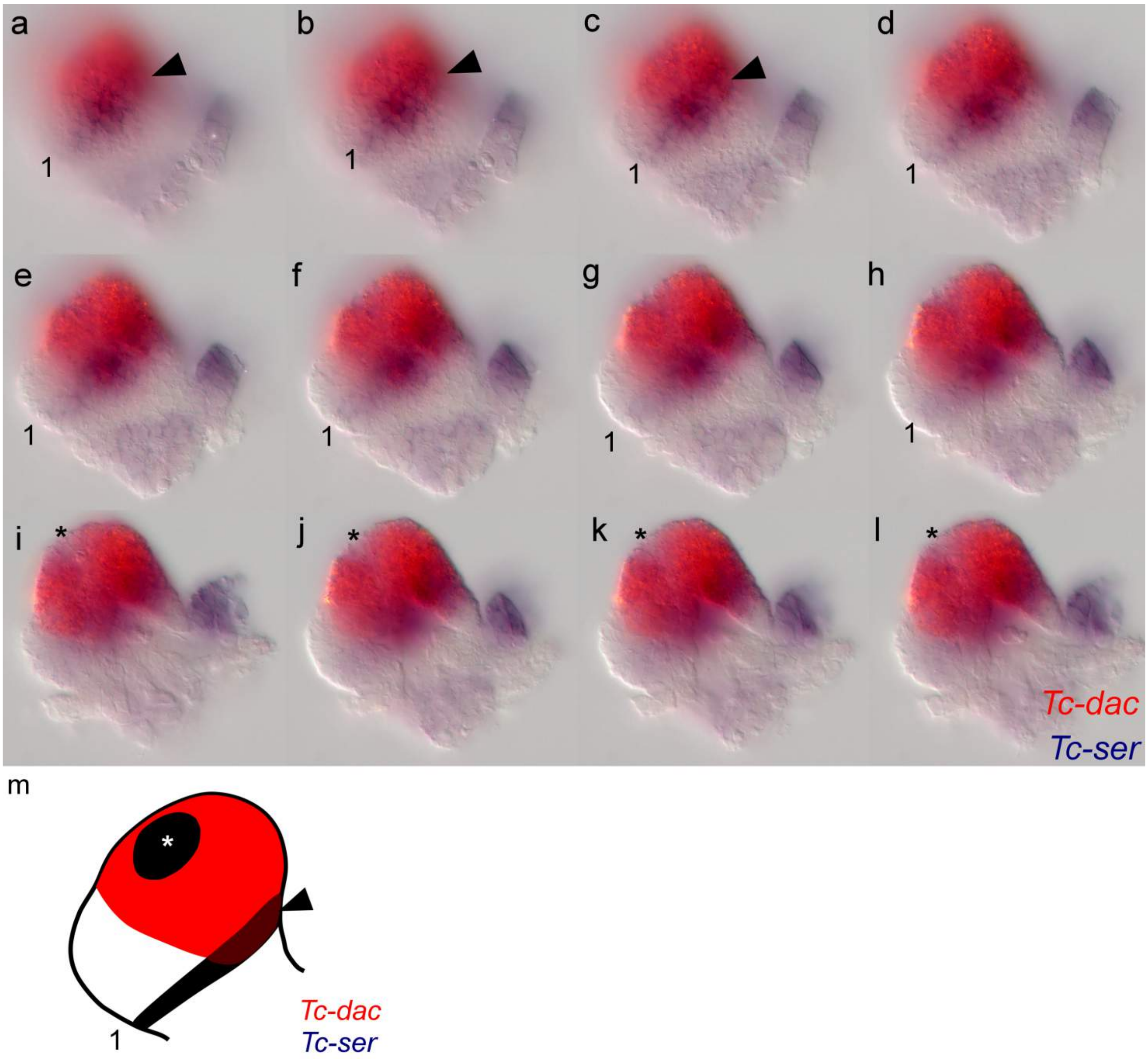

Supplementary figure S2: Montage of images of *Tc-ser* and *Tc-dac* expression in a dissected mandible with increasing depth of focus along the z axis. All views are lateral on the right, with medial on the left and distal on top. (a-l) *Tc-ser* (blue) and *Tc-dac* (red) gene expression was detected by *in situ* hybridisation. The plane of focus is highest in (a) where the surface of the lateral mandibular ectoderm is in focus. The focal plane incrementally descends through the dissected mandible from (a) through (l). An arrowhead indicates co-expression of *Tc-dac* and *Tc-ser* in the lateral ectoderm of the mandible in a-c and m. The spot domain of *Tc-ser* expression and corresponding gap in *Tc-dac* expression is indicated with an asterisk in (i-m). (m) Diagram showing the expression of Tc-dac and Tc-ser in the mandible. The *Tc-ser*-1 domain that marks the segmental boundary of the subcoxal segment is indicated with the number '1' where it is clear and in focus.

Supplementary Figure S3

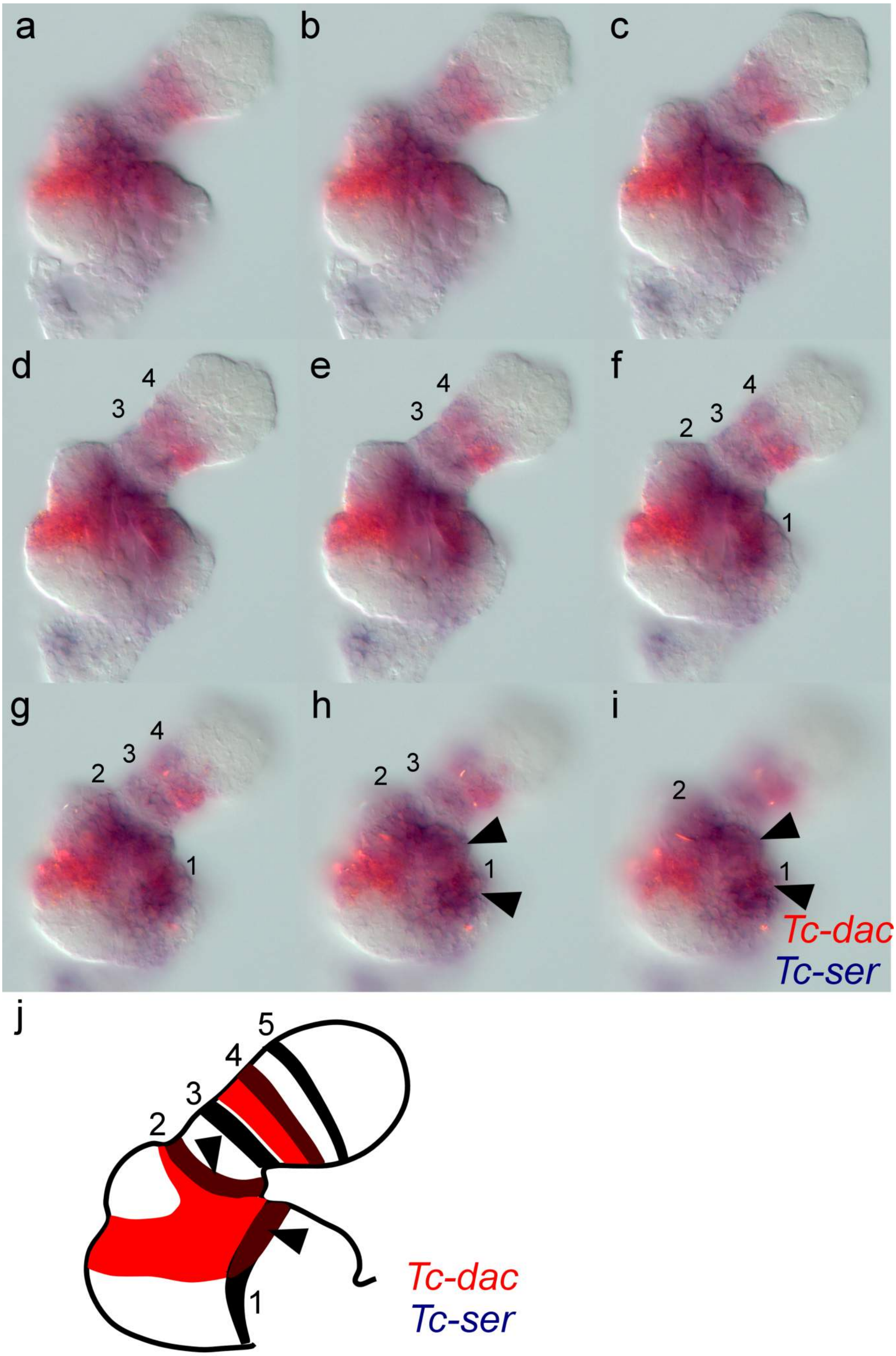

Supplementary fig. S3: Montage of images of *Tc-ser* and *Tc-dac* expression in a dissected maxilla with increasing depth of focus along the z axis. All views are lateral on the right, with medial on the left and distal on top. (a-i) *Tc-ser* (blue) and *Tc-dac* (red) gene expression was detected by *in situ* hybridisation. The plane of focus is highest in (a) where the surface of the ectoderm of the maxillary palp is in focus. The focal plane incrementally descends through the dissected maxilla from (a) through (i). (j) Diagram showing the expression of *Tc-dac* and *Tc-ser* in the maxilla. *Tc-ser* domains that mark segmental development are indicated with numbers where they are clear and that region of the ectoderm is in focus. Co-expression of *Tc-dac* and *Tc-ser* in the Tc-ser-1 and Tc-ser-2 domains is indicated with arrowheads in (h-i).

Supplementary Figure S4

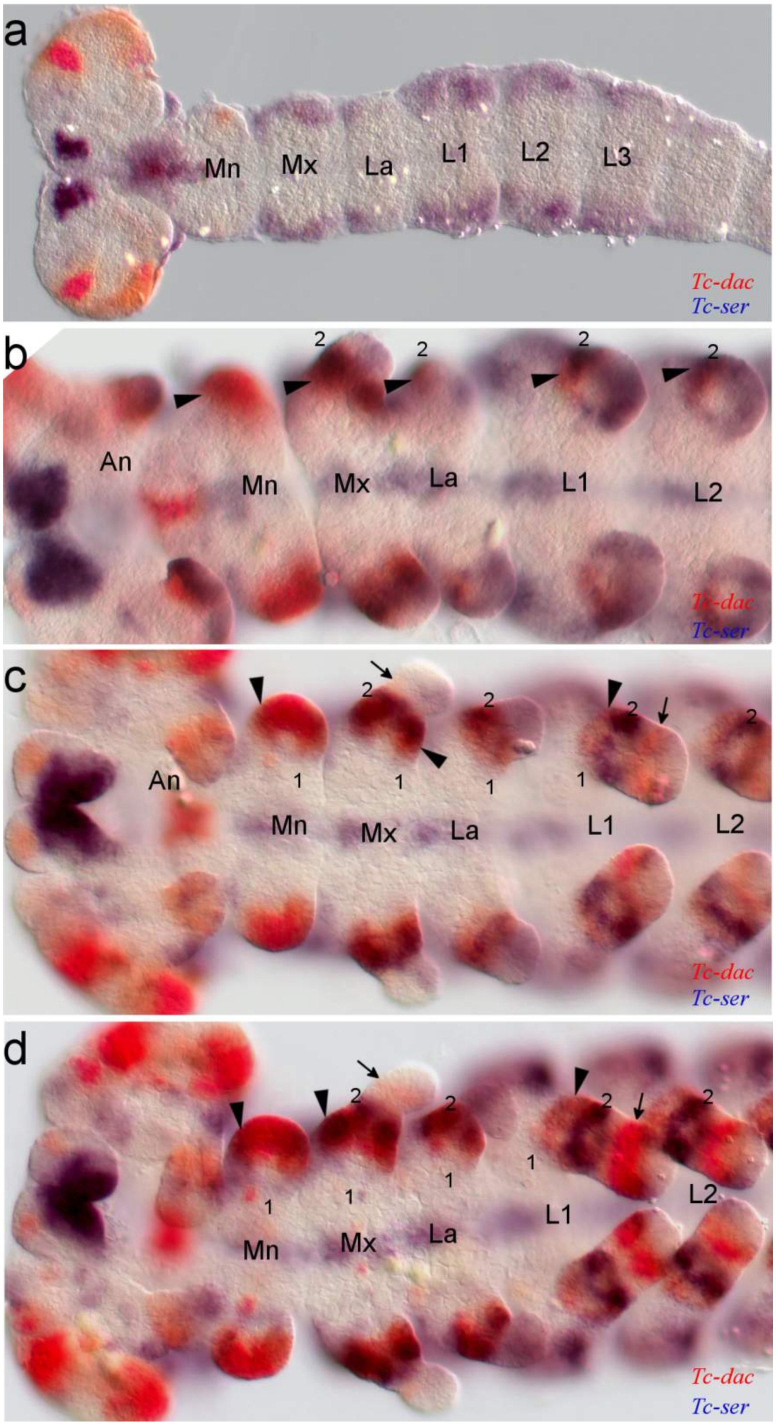

Supplementary Fig. S4: Serial homology of the subcoxa and coxa revealed by early expression domains of *Tc-dac* and *Tc-ser*. The Tc-ser coxal-2 expression domain is the first to be activated and appears simultaneously in all post-antennal appendages (except the mandible), co-expressed with *Tc-dac* (see Supplementary Fig. S4b and Supplementary Fig. S5a). The subcoxal-1 domain of *Tc-ser* domain subsequently appears simultaneously in all post-antennal limbs (including the mandible). All views are ventral with anterior to the left. *Tc-ser* (blue) and *Tc-dac* (red) gene expression was detected by *in situ* hybridisation. (a) Germ band extending embryo as the limb buds are forming. Faint *Tc-dac* expression is visible in the mandibular and maxillary segments. (b) Germ band extending embryo after the limb buds have formed. The coxal-2 domain of *Tc-ser* expression is present in all limb buds except the mandible and is co-expressed with the proximal domain of *Tc-dac* (arrowhead) which is expressed in all post-antennal appendages. (c) The subcoxal-1 domain of *Tc-ser* appears more proximal to the coxal-2 domain in all post-antennal appendages and is also at least partially co-expressed with the *Tc-dac* proximal domain. In the legs and maxilla, the distal domain of *Tc-dac* (arrow) is expressed along with a distal *Tc-ser* ring domain. (d) Fully germ band extended embryo. The distal domain of *Tc-dac* is more prominent than at earlier stages in the legs and the maxilla (arrow). Mandibular (Mn), maxillary (Mn), labial (La), leg (L1, L2), and antennal (An) segments are indicated.

Supplementary Figure S5

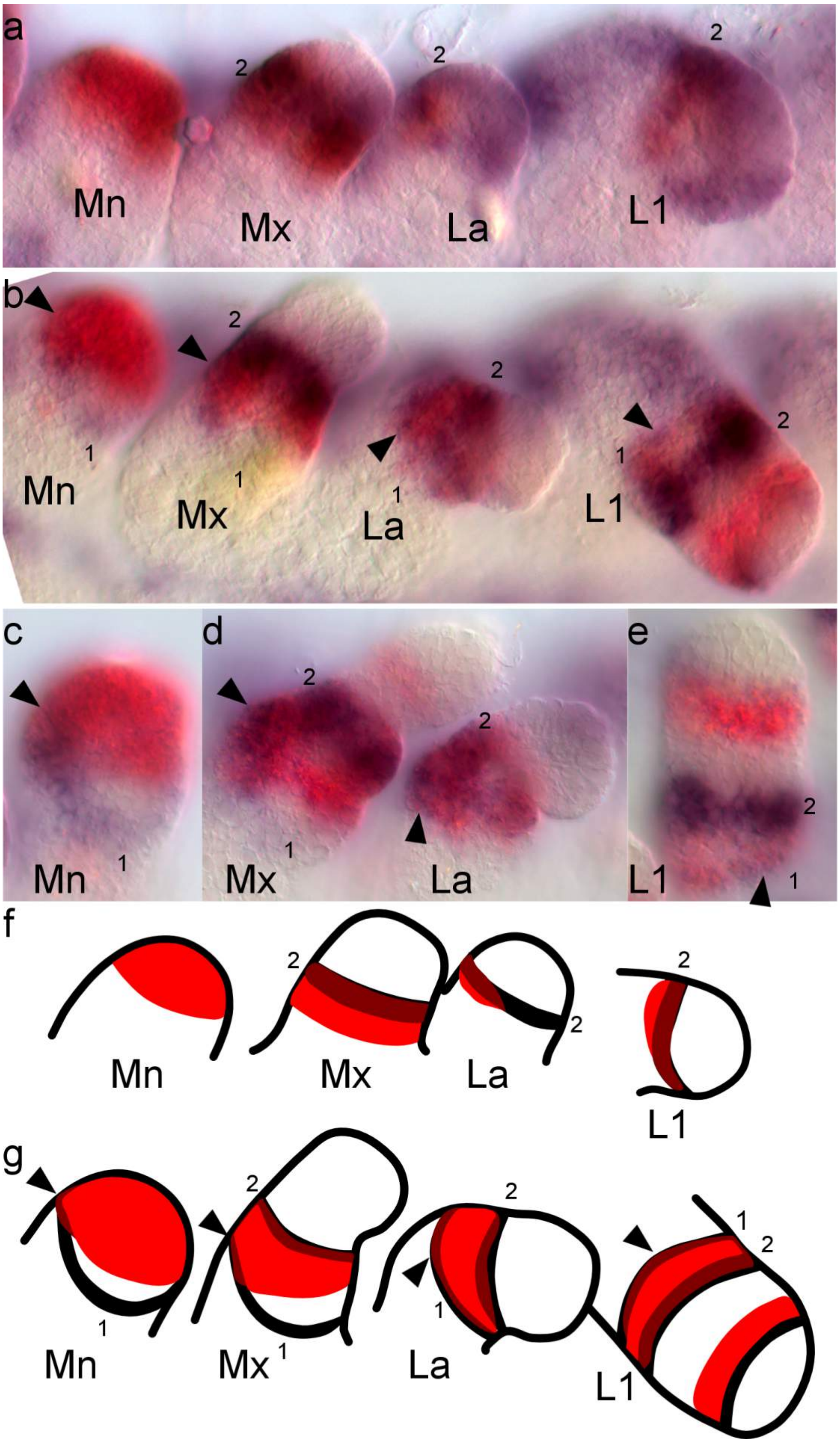

Supplementary Fig. S5: Onset of early expression domains of *Tc-ser* suggests serial homology of subcoxa and coxa between different appendages. Images show higher magnification of the expression of *Tc-ser* (blue) and *Tc-dac* (red) from supplementary Fig. S4. Gene expression was detected by *in situ* hybridisation. The first and second *Tc-ser* ring domains are interpreted to represent the distal segment boundaries of the subcoxa and the coxa respectively. Successive germ band extending stage embryos are shown (in a-e). All views are ventral with anterior to the left. (a) The proximal domain of *Tc-dac* appears first in each appendage. The maxilla, labial and leg limb buds have a coxal-2 domain of *Tc-ser* expression co-expressed with *Tc-dac* (indicated by the number 2). There is no domain of *Tc-ser* in the mandibular segment at this stage. (b) The subcoxal-1 domain of *Tc-ser* appears in all appendages and is co-expressed (indicated with an arrowhead) with the proximal domain of *Tc-dac* in later germ band extending embryo. The distal domain of *Tc-dac* is visible in the leg. The proximal domain of *Tc-dac* is more strongly expressed in the gnathal appendages. (c-e) The subcoxal-1 domain of *Tc-ser* expression is more clearly present in all post-antennal appendages in germ band retracting stage embryos in the mandible (c), maxilla (d) and first leg segment (e). (f) Diagrammatic representation of a) showing the onset of expression of the *Tc-ser* coxal-2 domain with the proximal domain of *Tc-dac.* (g) Diagrammatic representation of b) showing the onset of expression of the *Tc-ser* subcoxal-1 domain. Co-expression of the subcoxal -1 domain of *Tc-ser* with *Tc-dac* is indicated with an arrowhead.

Supplementary Figure S6

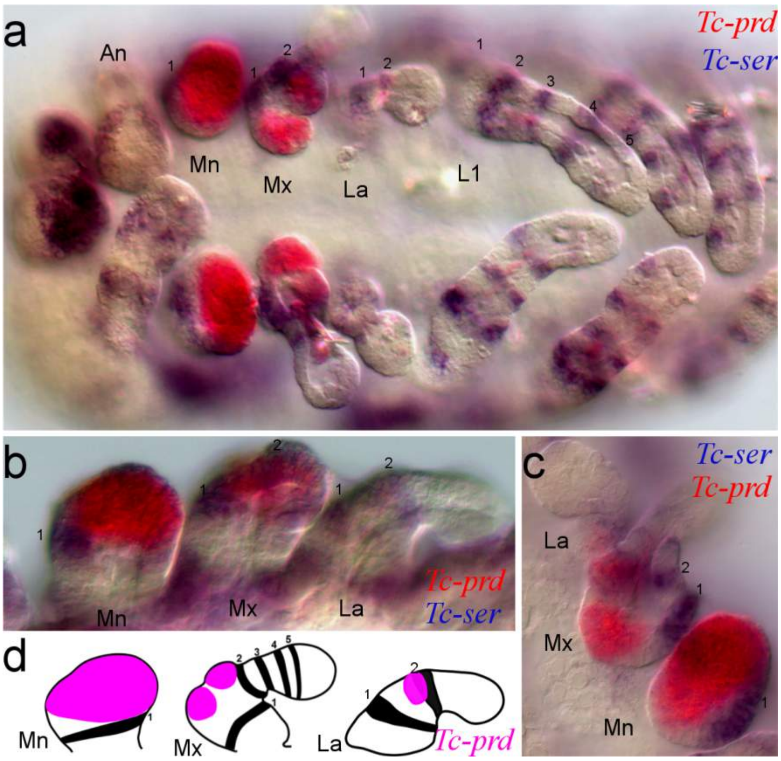

Supplementary Fig. S6: The endites develop in the coxal segment of the gnathal appendages. Gene expression was detected by *in situ* hybridisation. The developing endites are marked by *Tc-prd* expression in the gnathal appendages. *Tc-prd* expression is not present in the most proximal segment of the gnathal appendages, which supports serial homology of this segment between these limbs (a) *Tc-ser* (blue) and *Tc-prd* (red) expression in a fully retracted stage embryo. The five domains of *Tc-ser* expression are marked by arrows. Ventral view, anterior to the left. (b) Lateral view of gnathal appendages of a fully retracted *Tribolium* embryo. Expression of *Tc-prd* is distal to the *Tc-ser* subcoxal-1 domain and present in the second segment of the mandible, maxilla and labial appendages. (c) Ventral view, anterior is bottom. Again, expression of *Tc-prd* is distal to the *Tc-ser* subcoxal-1 domain and present in the second segment of the mandible and maxilla. (d) Diagram of *Tc-prd* (magenta) expression relative to the domains of *Tc-ser* expression in the mandibular, maxillary and labial appendages.

Supplementary Figure S7

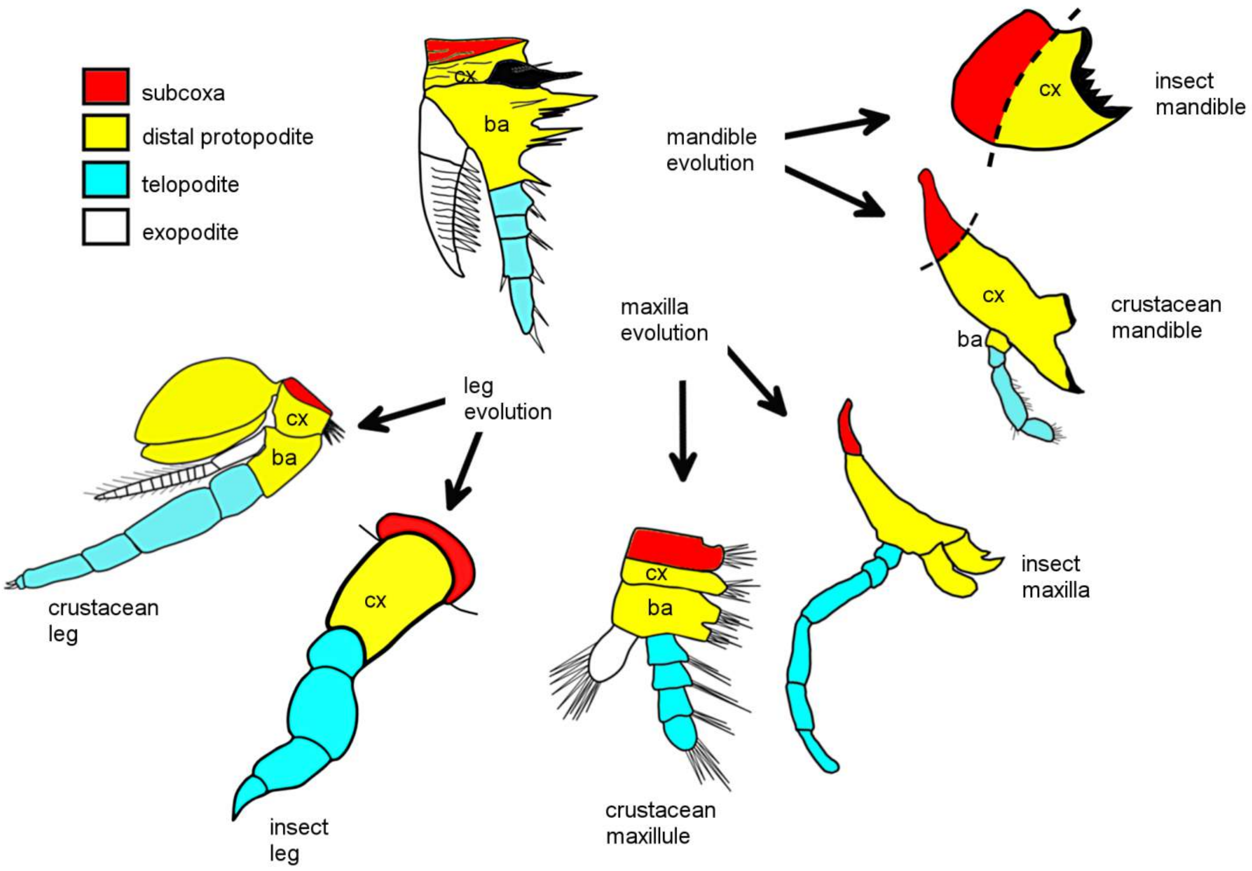

Supplementary Fig. S7: Evolutionary scenario of insect and crustacean appendage evolution from an ancestral biramous limb. Hypothetical evolution of three types of limb, the mandible, maxilla and leg are shown. The fossil record shows that arthropod post-antennal appendages evolved from serially homologous (albeit subtly differentiated) biramous limbs, which are present in Cambrian arthropods such as *Martinssonia elongata*. The biramous limb depicted here is from *M. elongata* and was adapted from[1]. The ancestral biramous limb was composed of two limb axes, an exopodite-derived ramus and a telopodite-derived ramus, which were connected to the protopodite, which is located at the base of the appendage. Insect limbs have evolved from the ancestral biramous limb through likely intermediary limb types present in extant crustacean species. The telopodite is highlighted in light blue, the exopodite is white. The protopodite is highlighted in yellow for the distal part and in red for the proximal part, the subcoxa. The protopodite is defined morphologically by the basal part of the limb to which the telopodite and exopodite are attached. Insect mandibles have lost both the telopodite and exopodites. The insect maxilla has lost the exopodites. It is hypothesized that maxillary stipes segment has formed from fusion of the coxa and basis which is present in more primitive hexapod maxillae. The insect leg has lost the exopodite. The subcoxal segment is present in each limb type as the most proximal segment (highlighted in red). The distal protopodite segments are labelled using crustacean terminology coxa (cx) or basis (ba) where homologous relationships are simpler to infer.

1 Müller, K. J. & Walossek, D. *Martinssonia elongata gen. et sp. n.*, a crustacean-like euarthropod from the Upper Cambrian 'Orsten' of Sweden. *Zool Scrip* **15**, 73-92, doi:10.1111/j.1463-6409.1986.tb00211.x (1986).
